# Executive Summary of Evidence-Based Guidelines for the Diagnosis and Treatment of Pediatric CKD-Mineral and Bone Disorder (Version 2024)

**DOI:** 10.1016/j.ekir.2026.106493

**Published:** 2026-06-03

**Authors:** Mo Wang, Hua-Ying Xiong, Chunhua Zhu, Xinyi Yu, Jia Jiao, Liwen Tan, Ying Shen, Hong Xu, Jianhua Mao, Jianhua Zhou, Xiao-yun Jiang, Qian Shen, Fang Wang, Songming Huang, Wenyan Huang, Zheng-kun Xia, Xiao-rong Liu, Hui Wang, Yu-bin Wu, Li Yu, Yu-hong Tao, Chun-lin Gao, Ying-jie Li, Shu-Zhen Sun, Dong-feng Zhang, Xiao-shan Shao, Li-Jun Zhao, Xiaowen Wang, Xi-qiang Dang, Hui-mei Huang, Yaolong Chen, Qiu Li, Aihua Zhang, Subspecialty Group of Nephrology, the Society of Pediatrics, Chinese Medical Association

**Affiliations:** 1 Department of Nephrology, Children’s Hospital of Chongqing Medical University, Chongqing, China; 2 National Clinical Research Center for Children and Adolescents’ Health and Diseases, Chongqing, China; 3 Department of Pediatric Nephrology, Children’s Hospital of Nanjing Medical University, Nanjing, China; 4 Department of Nephrology, Beijing Children’s Hospital, Capital Medical University, Beijing, China; 5 Department of Nephrology, Children’s Hospital of Fudan University, Shanghai, China; 6 Department of Nephrology, Children’s Hospital, Zhejiang University School of Medicine, Hangzhou, China; 7 Department of Pediatrics, Tongji Hospital, Tongji Medical College, Huazhong University of Science & Technology, Wuhan, China; 8 Department of Pediatric Rheumatology and Nephrology, Sun Yat-sen University First Affiliated Hospital, Guangzhou, China; 9 Department of Pediatrics, Peking University First Hospital, Beijing, China; 10 Department of Nephrology, Rheumatology and Immunology, Shanghai Children's Hospital, Shanghai Jiao Tong University, Shanghai, China; 11 Department of Pediatrics, Jinling Hospital, School of Medicine, Nanjing University, Nanjing, China; 12 Department of Pediatric Nephrology, Shengjing Hospital of China Medical University, Shenyang, China; 13 Department of Pediatrics, Guangzhou First People’s Hospital, Guangzhou, China; 14 Department of Pediatric Nephrology, West China Second University Hospital of Sichuan University, Chengdu, China; 15 Department of Nephrology, Guangzhou Women and Children's Medical Center, Guangzhou, China; 16 Department of Pediatrics, Shandong Provincial Hospital, Jinan, China; 17 Department of Nephrology and Immunology, Hebei Children’s Hospital, Shijiazhuang, China; 18 Department of Pediatric Rheumatology and Immunology, Guiyang Maternal and Child Health Hospital, Guiyang, China; 19 Department of Nephrology, Shanxi Children’s Hospital, Taiyuan, China; 20 Department of Nephrology, Wuhan Children’s Hospital, Wuhan, China; 21 Department of Pediatrics Nephrology, Second Xiangya Hospital of Central South University, Changsha, China; 22 Department of Nephrology, Xi’an Children’s Hospital, Xi’an, China; 23 The Centre of Evidence-based Social Science, School of Public Health, Lanzhou University, Lanzhou, China

**Keywords:** children, CKD-MBD, diagnosis, evaluation, guideline, treatment

## Abstract

Chronic Kidney Disease-Mineral and Bone Disorder (CKD-MBD) in children refers to the systemic mineral and bone metabolism disorders caused by CKD, including biochemical abnormalities, abnormalities in bone turnover, mineralization, quality, and ectopic calcification. Like adult patients, mineral metabolism and bone structure abnormalities are universally present in children with CKD. However, there have been no guidelines specifically for children in the past. The medication treatment and clinical practices for children are usually based on those for adults, thus lacking targeted guidance. Children are in the process of growth and development, and adult guidelines do not take into account the characteristics of children. In addition, the medication range for children is different from that of adults, and new drugs have been verified and tested in children. Therefore, based on the developmental characteristics of children, combined with expert opinions about adults, children, kidney, nutrition, and medicine, this guideline was initiated by the Subspecialty Group of Nephrology, the Society of Pediatrics, Chinese Medical Association. It answers many important clinical questions related to the diagnosis and treatment of pediatric CKD-MBD, aims to provide individualized treatment plans considering the clinical characteristics of children with CKD and their treatment goals, while addressing the needs of their growth and development.

CKD involves persistent kidney damage and functional decline.^1^ CKD-MBD is a systemic condition caused by CKD, including biochemical abnormalities, abnormalities in bone health (turnover, mineralization, and quality), and ectopic calcification.^2^ Globally, pediatric CKD prevalence ranges from 14.9 to 118.8 per million population, with end-stage renal disease affecting 4.9 to 38.7 per million.^3^ Mineral metabolism and bone abnormalities are nearly universal in children with CKD. As kidney function worsens, ectopic calcification becomes common, affecting 60% of children with end-stage renal disease, typically in vessels, lungs, kidneys, and heart.^4^^,^^5^ Children with CKD face the highest cardiovascular disease risk among their peers. Cardiovascular disease is strongly linked to severe complications and death, increasing mortality risk approximately 700-fold compared with children with CKD without cardiovascular disease.^6^ Because children are actively growing, CKD-MBD problems can manifest even before kidney failure occurs. Managing CKD-MBD is therefore critical for the long-term health of children with CKD.

Furthermore, the unique growth and developmental needs of children necessitate specific diagnostic and treatment approaches for CKD-MBD. Recognizing this, the Subspecialty Group of Nephrology, Society of Pediatrics (Chinese Medical Association) developed the 2024 evidence-based guideline. Its goals are to address key clinical concerns, improve comprehensive pediatric CKD management in China, and provide individualized treatment plans. These plans take into account children's clinical characteristics, treatment goals, and developmental needs. The guideline targets children aged 0 to 18 years with CKD (except for posttransplant recipients) and is intended for use by clinical physicians, pharmacists, and laboratory staff across health care institutions. To clarify the specific applicability of these recommendations within the Chinese pediatric context and to highlight how our evidence-based approach addresses unique developmental needs distinct from existing international standards, we summarize the generalizability and key differences between this guideline and other major global guidelines in Table 1.^7^, ^8^, ^9^, ^10^, ^11^, ^12^, ^13^

**Table 1:** Contextual applicability and comparison with international guidelines

No.	Clinical question	Recommendations	Note	Comparisons with other guidelines
1	Which serum biochemical indexes can be used to diagnose chronic kidney disease–mineral and bone disorder (CKD-MBD) in children?	1.1 We recommend serum calcium, phosphorus, alkaline phosphatase, intact parathyroid hormone (iPTH), and 25-hydroxyvitamin D_3_ (25(OH)D_3_) as the biochemical indicators for diagnosing CKD-MBD in children with CKD G2–G5D (1B). 1.2 We recommend monitoring the above-mentioned biochemical indicators in children with CKD from stage 2; and adjusting the frequency of testing based on the stage of CKD, rate of progression, and medication treatment during the G2-G5D period (1B).	In China, the implementation of this monitoring strategy is primarily constrained by testing capacity and health insurance coverage. Although calcium and phosphorus tests are widely available, primary hospitals often lack the facilities for iPTH and vitamin D testing, necessitating frequent referrals to higher-level hospitals. In addition, because these tests are mostly out-of-pocket costs, long-term monitoring imposes a significant financial burden on most families, and limited outpatient reimbursement may reduce patient adherence.	Our guidelines align with international consensus on core principles (selecting calcium, phosphorus, PTH, and alkaline phosphatase as key indicators, with frequency increasing as CKD progresses).^7^, ^8^, ^9^ However, 2 key differences exist: we recommend initiating monitoring earlier (from CKD stage 2) and include 25(OH)D_3_ as a routine indicator with specified frequencies, whereas Japanese Society for Dialysis Therapy (JSTD) guideline^9^ typically start at stage 3 and focus less on vitamin D.
2	How to evaluate linear growth disorder as one of the characteristic manifestations of CKD-MBD in children?	2.1 We suggested that infants aged 0–1 yrs with CKD G2-G5D should have their length measured at least once a month, and children aged 1–3 yrs should have their linear growth evaluated at least every 3 mos. Children aged > 3 yrs should have their linear growth evaluated at least every 6–12 mos (Not graded).	Regular height monitoring offers benefits that clearly outweigh its risks, enabling early detection of growth disorders through non-invasive, cost-free, and highly feasible measurements. Access is unaffected by economic or regional disparities, ensuring equitable health outcomes for all children with CKD.	Our guidelines align with the international consensus that younger age necessitates more frequent monitoring.^9^, ^10^, ^11^, ^12^ The primary distinction is that other guidelines (e.g., ERA-EDTA) implement more detailed stratification by CKD stage, whereas ours prioritize clinical practicality by unifying recommendations for all stages into 3 age-specific intervals.
3	Which biochemical indicators can be used to assess the nature and severity of bone abnormalities in children with CKD-MBD?	3.1 In children with CKD G3a–G5D, it is recommended to monitor iPTH and bone alkaline phosphatase together to assess the nature and severity of bone abnormalities (1B). 3.2 Based on the characteristics of children's growth and development and the disease features of pediatric CKD-MBD, measurement of C-terminal FGF23 (cFGF23) may be considered as an early indicator of mineral metabolism disturbances in settings where this assay is available. (2B).	In China, the generalizability of this monitoring strategy is primarily constrained by testing accessibility and financial burden. As a novel biomarker, FGF23 are available only in a few tertiary hospitals and are entirely out-of-pocket with high costs. The inability of primary hospitals to perform these tests necessitates frequent referrals, further increasing health care expenses for families.	Our guidelines share core principles with ERA-EDTA^10^ and Kidney Disease: Improving Global Outcomes (KDIGO) guideline^7^ (using Ca, P, alkaline phosphatase, PTH as key indicators and emphasizing trends over single values). However, our approach is more pediatric-specific: we recommend bone alkaline phosphatase and early FGF23 monitoring to assess bone abnormalities and growth, whereas ERA-EDTA^10^ focuses on conventional markers and KDIGO^7^ advises against routine bone turnover markers.
4	What are the appropriate imaging techniques for detecting changes in bone mineral density (BMD) and bone mass in children with CKD-MBD?	4.1 It is reasonable to consider dual-energy x-ray absorptiometry (DXA) for bone mineral density to assess bone quality and fracture risk for children with CKD G3a–G5D (2C).	In China, DXA testing is highly generalizable. The equipment is widely available in hospitals at all levels, with low costs largely covered by medical insurance, ensuring good health equity. Although technical limitations exist in assessing bone mineral density in children with poor growth, the overall benefits clearly outweigh the risks.	Our guideline aligns with KDIGO guideline^7^ on using DXA to assess fracture risk in advanced CKD but differs in population focus (children vs. adults) and purpose (pediatric-specific bone strength assessment vs. treatment guidance).
5	Is bone histological examination necessary for children with CKD-MBD?	5.1 If it is necessary to determine or adjust treatment decisions by understanding the type of renal osteodystrophy, bone biopsy might be performed for children with CKD G3a–G5D (2C).	In China, the generalizability of bone biopsy for children with CKD-MBD is severely limited. Due to its invasive nature, high cost, and technical complexity, it is currently performed only in a few national pediatric nephrology centers and is difficult to implement at the primary level.	Our guideline aligns with the KDIGO adult guideline^7^ in emphasizing that bone biopsy should be guided by treatment decision-making needs. The difference lies in our specific focus on children with a Grade 2C recommendation, whereas KDIGO^7^ targets adults and is not graded.
6	How to detect vascular calcification in children with CKD-MBD.	6.1 For selected children with CKD G3a–G5D at high risk for cardiovascular complications, computed tomography (CT) examination may be considered to assess coronary artery and abdominal aortic calcification when the results are expected to meaningfully influence clinical management. However, CT is not recommended for routine screening because of radiation exposure concerns (2D). 6.2 In children with CKD G3a–G5D, lateral abdominal radiograph and echocardiogram can be used to detect the presence or absence of valvular calcification. These are reasonable and preferred alternatives to computed tomography-based imaging in most clinical situations (Not graded).	In China, the generalizability of this imaging assessment is limited by CT's radiation exposure, high cost, and operational difficulty in children. CT equipment is mainly concentrated in tertiary hospitals. Children often have difficulty cooperating during scans, necessitating sedation or anesthesia, which increases complexity and medical costs. In addition, limited insurance reimbursement for CT may increase the cost on families. In contrast, lateral abdominal X-rays and echocardiography—being radiation-free, low-cost, and widely available, are more suitable alternatives for routine pediatric screening.	Our guidelines are highly consistent with the 2017 KDIGO guidelines^7^ in core principles. The differences lie in the following: KDIGO^7^ further defines patients with known calcification as being at highest cardiovascular risk (2A) and suggests to guide CKD-MBD management; whereas our guidelines focus more on the specificity of the pediatric population, emphasizing that CT should only be considered in high-risk children when results are expected to meaningfully influence clinical decisions, and its use is strictly limited due to radiation concerns.
7	What are the normal ranges of serum calcium and phosphorus in children with CKD G2–G5D at different ages?	7.1 We suggest that for children with CKD G2–G5D, to maintain serum calcium and phosphorus within the normal range of the corresponding age (Not graded).	In China, maintaining serum calcium and phosphorus within the normal range for children with CKD is highly generalizable. Serum calcium and phosphorus testing is widely available at the primary level as routine tests, and basic medications such as phosphate binders are covered by medical insurance, resulting in a relatively low financial burden for families.	Our guidelines align with international guidelines^7^^,^^9^^,^^11^ in emphasizing the maintenance of calcium and phosphorus within age-appropriate normal ranges. The difference lies in approach: KDIGO^7^ and others provide fixed reference values, whereas we recommend referring to each laboratory's own normal ranges to accommodate methodological differences, offering greater clinical operability.
8	What is the recommended range of daily intake of dietary calcium and phosphorus for children with CKD G3a–G5D?	8.1 We suggest that the total daily intake of elemental calcium (including phosphate binders) in children with CKD G2-G5D should not exceed twice the Dietary Reference Intakes (DRI) (Not graded). 8.2 We suggest that children with CKD G3a–G5D should not exceed their daily DRI intake of phosphorus while ensuring protein intake. For children with elevated levels of serum phosphorus and iPTH, it is recommended that the intake of dietary phosphorus should not exceed 80% of DRI at the same age (Not graded).	In China, this nutritional intervention has good generalizability. With the expansion of clinical nutrition departments and the development of telemedicine, access to professional dietary guidance is continuously improving. The dietary guidance itself incurs no additional costs, as families can acquire the necessary skills through health education.	Our guidelines align with international guidelines^11^^,^^13^ in using DRI as the basis for managing calcium and phosphorus intake, with consistent core principles. The difference lies in the approach: Pediatric Renal Nutrition Taskforce^13^ and K/DOQI^11^ provide age-specific numerical values, whereas we adopt DRI percentages, which offers greater clinical operability and adaptability to updates in nutritional guidelines.
9	What is the appropriate calcium supplementation strategy for hypocalcemia in children with CKD G3a–G5D?	For the children with CKD G3a–G5D, when dietary intake is insufficient to meet the DRI requirements and/or when hypocalcemia is concomitantly present, the administration of calcium supplementation is recommended (1C).	In China, oral calcium supplements, as basic medications, are widely covered by medical insurance and available at primary institutions, imposing a low financial burden on families. Serum calcium testing can be performed at all hospital levels, providing reliable evidence for clinical decision-making.	Our guidelines align with the KDIGO guidelines^7^ in aiming to maintain serum calcium within the age-appropriate normal range. The difference lies in our specification of 2 clear indications for initiating calcium supplementation (dietary intake below DRI requirements or presence of hypocalcemia).
10	How to choose phosphate-lowering drugs for children with CKD G3a–5D who develop hyperphosphatemia	10.1 For the children with CKD G3a–G5D and serum calcium levels below the age-specific normal upper limit, it is recommended to use calcium-based phosphate binders (1B) and/or combined with noncalcium metal-based phosphate binders (1B), nonmetal-based phosphate binders (1A) for the treatment of hyperphosphatemia. 10.2 If the child has comorbidities such as persistent hypercalcemia or arterial calcification, low iPTH levels, or dystrophic bone disease, the use of calcium-based phosphate binders should be restricted (1B).	In China, this intervention has good generalizability. Calcium-based phosphate binders are widely covered by medical insurance and available at primary medical institutions. Novel noncalcium-based phosphate binders, with some included in the national insurance negotiation catalog, are increasingly accessible. Relevant laboratory tests are available at all hospital levels, supporting individualized treatment decisions.	Our guidelines align with KDIGO^7^ in emphasizing that the choice of phosphate binders should be individualized based on clinical conditions such as serum calcium, vascular calcification, and bone disease. The difference lies in our explicit recommendation of combination/monotherapy regimens for 3 categories of phosphate binders with evidence grading, and our detailed listing of 4 specific clinical scenarios warranting restriction of calcium-based binders.
11	How to adjust the dialysis regimen for hypocalcemia or hyperphosphatemia in children with CKD G5D	11.1 In the children with CKD G5D, when the ionized calcium concentration falls below 1.25 mmol/l, individualized adjustments to the calcium-containing dialysate prescription should be considered. Close monitoring of serum calcium levels during treatment is advisable (Not graded). 11.2 For the children with persistent hyperphosphatemia in CKD G5D, implementing an intensified dialysis regimen may be considered to enhance the removal of phosphate from the blood (2D).	In China, the generalizability of intensive dialysis regimens for children faces multiple challenges. The regimen still lacks support from large-sample studies and is associated with high costs and significant time commitments, heavily relying on families' economic status. Because of the uneven distribution of pediatric-specific dialysis resources, its feasibility is currently limited, hindering widespread implementation and potentially posing challenges to health equity.	Our guidelines align with KDIGO^7^ and KDOQI^11^ in recommending adjustments to dialysate calcium concentration and enhanced phosphate removal. The key differences are as follows: we are the first to propose a specific trigger threshold for initiating calcium adjustment in children (ionized calcium < 1.25 mmol/l), and assign a Grade 2D recommendation to intensive dialysis regimens, reflecting a cautious approach to pediatric evidence.
12	What is the recommended range of serum vitamin D concentration for the children with CKD G2–G5D?	12.1 We recommend that the serum 25(OH)D concentration for the children with CKD G2–G5D should be maintained above 30 ng/ml (> 75 nmol/l) (Not graded). 12.2 We recommend supplementing natural vitamin D for children with CKD G2–G5D who have serum 25(OH)D levels < 30 ng/ml, and selecting the appropriate regimen based on age and severity of deficiency (Not graded).	In China, this intervention has high generalizability. 25(OH)D testing is widely available, and natural vitamin D supplements are low-cost with broad insurance coverage. Vitamin D supplementation has become routine in pediatric practice.	Our guidelines are highly consistent with the European Society for Paediatric Nephrology guidelines^12^ on the core principles of vitamin D supplementation, both using 30 ng/ml as the threshold and implementing stepwise supplementation based on age and severity of deficiency when levels fall below this value.
13	What is the recommended range for iPTH levels in the children with CKD G3a–G5D?	13.1 We suggest that for children with CKD G2 and G3, it is recommended to control iPTH levels within the normal range. For children with CKD G4, iPTH levels should be controlled below twice the upper limit of the normal range. For children with CKD G5 and G5D, it is recommended to maintain iPTH levels between 2 and 9 times the upper limit of the normal range (Not graded).	In China, the implementation of iPTH target management is highly feasible. iPTH testing is widely available at all hospital levels. Active vitamin D and its analogs, as first-line therapeutic agents, are included in the national medical insurance catalog, readily accessible at primary institutions, and impose a low financial burden on families.	Our guidelines align with KDIGO,^7^ JSDT,^9^ and KDOQI guideline^11^ in progressively widening iPTH targets as CKD advances. The difference lies in our adoption of 2–9 times upper limit of the normal range (consistent with KDIGO), which is broader than KDOQI and JSDT, offering greater inclusivity.
14	How to supplement active vitamin D and vitamin D analogues in children with CKD G3a-G5D when they have secondary hyperparathyroidism (SHPT)?	14.1 For children with CKD G3–G5D who have severe and progressive SHPT, we recommend starting initial oral calcitriol therapy to reduce excessively high iPTH levels (1B). 14.2 When the total dose of weekly oral calcitriol is consistent, oral calcitriol can be administered daily or intermittently, with an initial dose of 10–20 ng/kg/d, to control iPTH levels within a reasonable range (1B). 14.3 For children with mild SHPT or stable iPTH levels in CKD G3-G5D, we suggest oral treatment with vitamin D analogues. For CKD patients, we recommend oral administration of alfacalcidol (1C) and paricalcitol (1B) to control iPTH levels.	In China, the generalizability of this treatment strategy is favorable. First-line drugs such as calcitriol are widely covered by medical insurance and available at primary institutions. Newer vitamin D analogs are increasingly accessible. iPTH testing is widely available, providing reliable guidance for clinical dose adjustment.	Our guidelines align closely with international guidelines (KDIGO,^7^ JSDT,^9^ KDOQI^11^) in recommending active vitamin D and its analogs for SHPT treatment. The key differences are that we specify pediatric indications for initiating therapy (severe and progressive SHPT) and provide detailed dosing regimens (10–20 ng/kg/d, daily or intermittent oral administration), with stratified recommendations based on SHPT severity (mild vs. severe), offering greater pediatric-specific operability compared to other guidelines.
15	How to supplement calcimimetics in the children with CKD G3a–G5D when they have SHPT	15.1 The combination of cinacalcet, calcitriol, or vitamin D analogues could be considered for children with CKD G4–G5D who have persistent iPTH levels > 300 pg/ml in order to effectively control iPTH within the target range (2C). 15.2 The treatment for children with CKD G4–G5D and persistently elevated iPTH levels (> 300 pg/ml) could initiate cinacalcet therapy at a low dose (≤ 0.20 mg/kg/d), with subsequent dosage adjustments based on treatment efficacy, up to a maximum daily dose of 60 mg (2C).	In China, the generalizability of cinacalcet combination therapy faces challenges: lack of pediatric-specific formulations requires splitting adult tablets; medical insurance reimbursement is limited to adults in some regions, placing a financial burden on pediatric families; and primary hospitals have limited capacity for standardized monitoring of children with persistently elevated PTH levels.	Our guidelines align with KDIGO^7^ and JSDT guideline^9^ in recognizing the value of cinacalcet for controlling SHPT. The key difference is that we are the first to specify a pediatric threshold for initiating cinacalcet (iPTH > 300 pg/ml) and a starting dose (≤ 0.20 mg/kg/d), whereas KDIGO, citing insufficient pediatric evidence, only emphasizes caution regarding hypocalcemia without specific recommendations, and JSDT mentions its use but lacks pediatric-specific protocols.

### Methodological Approach

The flow diagram for developing this guideline has been given in Figure 1. The clinical questions were formulated through a rigorous multistep process: an initial pool of 25 questions generated from a literature review was refined by the steering committee and subsequently prioritized using a Delphi survey using a 3-point Likert scale, resulting in 15 final population, intervention, comparison, and outcome–structured questions. Comprehensive evidence searches were conducted across major Chinese and English databases and guideline repositories up to December 2023. Study selection and quality assessment were independently performed by 2 reviewers, using A Measurement Tool to Assess Systematic Reviews (AMSTAR) for systematic reviews, the Cochrane Collaboration’s tool for randomized controlled trials (RCTs), and the Newcastle-Ottawa Scale for observational studies, with disagreements resolved through discussion or steering committee consultation. Evidence synthesis followed a hierarchical strategy, prioritizing recent, high-quality systematic reviews (AMSTAR score ≥ 9); where such evidence was absent, biased, or inconsistent, high-quality RCTs or observational studies were sequentially evaluated and synthesized.

**Figure 1:**
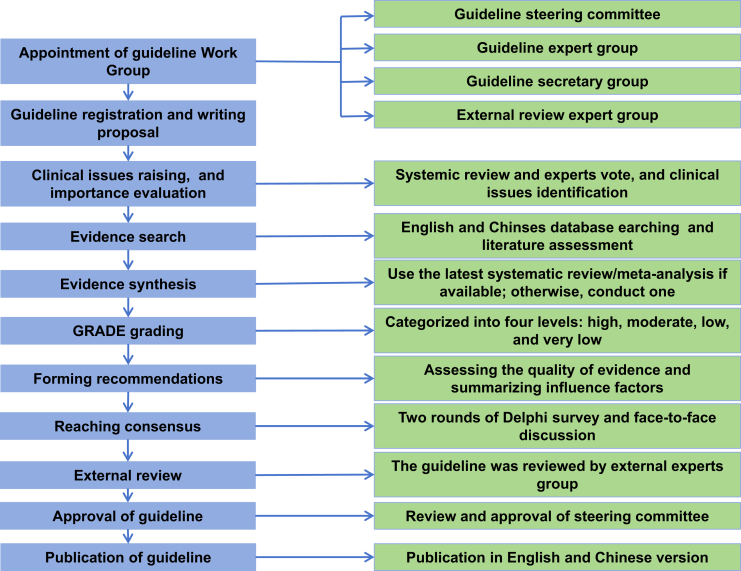
Flow diagram of developing this guideline. GRADE, Grading of Recommendations Assessment, Development, and Evaluation.

Recommendations were developed using the Grading of Recommendations Assessment, Development, and Evaluation system, integrating evidence quality with considerations of benefits, harms, patient values, and resource use.^14^ Consensus was established through 2 rounds of Delphi surveys supplemented by face-to-face discussions. A strict consensus rule was applied: recommendations required agreement from more than two-thirds of the voting experts. For items failing to reach this threshold, modifications were made based on expert feedback, followed by revoting until consensus was achieved. This process ensured robust guidance even in areas with weak evidence, culminating in final recommendations categorized as strong (Grade 1) or weak (Grade 2).

### Section 1: Assessment of Serum Biochemical Indexes and Linear Growth

1. We recommend serum calcium, phosphorus, alkaline phosphatase, intact parathyroid hormone (iPTH), and 25-hydroxyvitamin D_3_ (25(OH)D_3_) as the biochemical indicators for diagnosing CKD-MBD in children with CKD G2-G5D (1B).
2. We recommend monitoring the above-mentioned biochemical indicators in children with CKD from stage 2; and adjusting the frequency of testing based on the stage of CKD, rate of progression, and medication treatment during the G2-G5D period (1B).
3. We suggest that infants aged 0 to 1 years with CKD G2-G5D should have their length measured at least once a month, and children aged 1 to 3 years should have their linear growth evaluated at least every 3 months. Children aged > 3 years should have their linear growth evaluated at least every 6 to 12 months (Not graded).

### Rationale

CKD is associated with phosphorus retention and the development of hyperphosphatemia. Research has demonstrated that even in the early stages of CKD (G3),^15^ pediatric patients frequently exhibit abnormalities in calcium and phosphorus metabolism, including hypocalcemia (12.2%–49%), hyperphosphatemia (18.4%–65.9%), vitamin D deficiency (reduced serum 25(OH)D levels), and elevated iPTH.^16^^,^^17^ The 2017 KDIGO Clinical Practice guidelines^7^ recommend initiating monitoring of serum calcium, phosphorus, PTH, and alkaline phosphatase from CKD G2, whereas the European Pediatric Dialysis Working Group guidelines^8^ suggest beginning at G3. Considering that children with CKD G2-G3 already exhibit significant abnormalities in biochemical indicators, this guideline recommends initiating continuous monitoring of these indicators from G2 of CKD onward (Table 2) (Supplementary Tables S1–S5).

**Table 2:** Recommended frequency of biochemical indicator testing for children with CKD G2-5D

Parameter	CKD G2	CKD G3	CKD G4	CKD G5-G5D
Serum calcium	6 mos	6 mos	3 mos	1 mo
Serum phosphorus	6 mos	6 mos	3 mos	1 mo
ALP	12 mos	6 mos	3 mos	1–3 mos
iPTH	12 mos	6 mos	3 mos	1–3 mos
25(OH)D_3_	12 mos	6 mos	3 mos	1–3 mos

25-hydroxyvitamin D_3_, 25(OH)D_3_; ALP, alkaline phosphatase; CKD, chronic kidney disease; iPTH, intact parathyroid hormone.

Bone abnormalities in children with CKD-MBD can manifest as growth retardation, ostealgia (bone pain), skeletal deformities, epiphyseal slipping, and fractures. Among these, growth retardation and skeletal deformities are clinical manifestations specific to children.^18^ Early intervention is crucial for improving the final height of children with CKD and short stature. Therefore, regular assessment of skeletal growth is necessary, and the most fundamental method is measuring length/height.

Monitoring frequency is determined based on age and growth characteristics: infants aged 0 to 1 year with CKD stages G2 to G5D should have their height measured at least once a month; children aged 1 to 3 years should have their linear growth evaluated at least every 3 months; and children aged > 3 years should have their linear growth evaluated at least every 6 to 12 months. Monitoring frequency should be increased during infancy and adolescence because of the characteristic rapid growth during these periods.

### Section 2: Evaluation of Bone Abnormalities

1. In children with CKD G3a to G5D, it is recommended to monitor iPTH and bone alkaline phosphatase (BAP) together to assess the nature and severity of bone abnormalities (1B).
2. Based on the characteristics of children's growth and development and the disease features of pediatric CKD-MBD, measurement of C-terminal FGF23 may be considered as an early indicator of mineral metabolism disturbances in settings where this assay is available. (2B).
3. It is reasonable to consider dual-energy x-ray absorptiometry (DXA) for bone mineral density (BMD) to assess bone quality and fracture risk for children with CKD G3a to G5D (2C).
4. If it is necessary to determine or adjust treatment decisions by understanding the type of renal osteodystrophy, bone biopsy might be performed for children with CKD G3a to G5D (2C).

### Rationale

Bone metabolism process in CKD-MBD involves the cycle of bone formation and bone resorption. Current biochemical markers (e.g., iPTH, BAP, and FGF23) can reflect bone metabolic abnormalities; however, all have limitations. IPTH is commonly used clinically to assess bone turnover status.^19^ High levels (> 300 pg/ml) often indicate high-turnover bone disease; however, predictive efficiency is only 62%, whereas low levels (< 150 pg/ml) predict low-turnover bone disease with 83% efficiency.^20^ A German study showed that iPTH was significantly higher in high-turnover bone disease patients than in the non–high-turnover group (26 ± 18 vs. 8 ± 9 pmol/l, *P* < 0.001).^21^ BAP is a bone formation–specific marker; it outperforms iPTH (area under the curve = 0.824 vs. area under the curve = 0.563) in distinguishing low from non–low-bone turnover.^22^ FGF23 increases significantly with CKD progression (median levels: CKD G3: 128 kRU/L vs. CKD G5: 654 kRU/L).^17^ It serves as an early monitoring indicator but has limited clinical adoption (Supplementary Table S6–S10). The 2017 KDIGO guidelines recommend combining iPTH and BAP to assess bone disease.^7^ The emerging calcium isotope ratio technique may offer a new direction for noninvasive assessment.^23^

Abnormal BMD in children with CKD significantly increases fracture risk and may lead to skeletal deformities, chronic pain, and severe long-term sequelae in adulthood.^24^^,^^25^ DXA is recognized internationally as the gold standard for BMD assessment because of its low cost, minimal radiation, and accessibility.^26^ Research showed that compared with nondialysis children, the lumbar or hip BMD-Z scores in the dialysis-dependent group were significantly lower(*P* < 0.01), with >60% exhibiting reduced bone mineral content and density.^27^ The lumbar BMD-Z scores were significantly negatively correlated with iPTH (*r* = −0.47) and serum phosphorus (*r* = −0.61).^28^ However, DXA relies on areal density, cannot distinguish trabecular from cortical bone damage, and low Z-scores in short-stature children may reflect growth impairment rather than true bone deficits.^29^ Z-scores must be corrected using height or apparent bone density (corrected Z-scores increased from −0.03 to 0.49).^30^ The guidelines recommend DXA for fracture risk assessment in children with CKD G3a to G5D, requiring weighing radiation risks against benefits and mandatory Z-score correction.^7^^,^^10^ Large-scale studies are needed to validate DXA's predictive value.

Except for BMD, bone histomorphometry remains the gold standard for evaluating CKD-MBD bone disease, accurately assessing bone turnover, mineralization, and volume. Studies indicate that approximately half of pediatric patients with CKD exhibit high-turnover bone disease, whereas low-turnover disease historically accounted for 10% to 20%.^31^, ^32^, ^33^ However, recent studies (e.g., a 2020 Brazilian study of 42 dialysis children) show a significant increase in low-turnover disease (59.5%),^34^ potentially linked to widespread calcitriol/vitamin D analog use. Beyond turnover abnormalities, mineralization defects are highly prevalent in children with CKD. A 2012 US study found mineralization defects in all pediatric patients with CKD G2 to G5 (*n* = 52), with prevalence increasing with CKD progression.^35^ And a 2010 study reported that 48% of dialysis children (*n* = 161) had mineralization defects^36^ (Supplementary Table S16–S20).

Although bone biopsy guides targeted treatment for refractory hypercalcemia, unexplained hypophosphatemia, deformities, and fractures, its invasive nature, cost, and need for specialized operators limit routine use. In KDIGO 2017 guidelines, bone biopsy should be considered for children with CKD G3a to G5D only when unexplained bone disease or renal osteodystrophy type affects treatment decisions.^37^ Future pediatric research is needed to correlate noninvasive biomarkers with histomorphometric findings.

### Section 3: Detection of Vascular Calcification

1. For selected children with CKD G3a to G5D at high risk for cardiovascular complications, computed tomography (CT) examination may be considered to assess coronary artery and abdominal aortic calcification when the results are expected to meaningfully influence clinical management. However, CT is not recommended for routine screening due to radiation exposure concerns (2D).
2. In children with CKD G3a to G5D, lateral abdominal radiograph and echocardiogram can be used to detect the presence or absence of valvular calcification. These are reasonable and preferred alternatives to CT-based imaging in most clinical situations (not graded).

### Rationale

Extrarenal calcification is prevalent in patients with CKD, affecting ≤60% of children with end-stage renal disease.^4^^,^^5^^,^^38^A UK multicenter study revealed coronary artery calcification in 10% of pediatric patients with end-stage renal disease, with higher prevalence in the dialysis group (9/77 vs. 1/23).^39^ After a median follow-up of 1.45 years, coronary artery calcification scores increased from 8.1 to 42.61, indicating rapid calcification progression.^40^

Evidence in adults shows an abdominal aortic calcification prevalence of 68.5% (CT detection rate: 84.9% > X-ray: 65.2%).^41^ A Chinese study found valvular calcification in 77.4% of dialysis patients^42^ (Supplementary Tables S21–S25). Among imaging modalities, CT offers optimal sensitivity and stability, albeit at a higher cost. The KDIGO 2017 guidelines recommend lateral abdominal X-ray (for vascular calcification) and echocardiography (for valvular calcification) as practical alternatives-echocardiography being radiation-free, cost-effective, and reasonably sensitive, whereas X-ray provides excellent cost-effectiveness.^7^

However, significant evidence gaps exist in pediatric populations; the applicability of echocardiography for detecting valvular calcification and X-ray for assessing vascular calcification remains unvalidated in children and requires further investigation.

### Section 4: Management of Serum Calcium and Phosphorus Levels

1. We suggest that children with CKD G2 to G5D maintain serum calcium and phosphorus within the normal range of the corresponding age (Not graded).
2. We suggest that the total daily intake of elemental calcium (including phosphate binders) in children with CKD G2 to G5D should not exceed twice the Dietary Reference Intakes (DRI) (Not graded).
3. We suggest that children with CKD G3a to G5D should not exceed their daily DRI intake of phosphorus while ensuring protein intake. For children with elevated levels of serum phosphorus and iPTH, it is recommended that the intake of dietary phosphorus should not exceed 80% of DRI at the same age (Not graded).
4. For children with CKD G3a to G5D, when dietary intake is insufficient to meet the DRI requirements and/or when hypocalcemia is concomitantly present, the administration of calcium supplementation is recommended (1C).
5. For children with CKD G3a to G5D and serum calcium levels below the age-specific normal upper limit, it is recommended to use calcium-based phosphate binders (1B) and/or combined with noncalcium metal-based phosphate binders (1B), nonmetal–based phosphate binders (1A) for the treatment of hyperphosphatemia.
6. If the child has comorbidities such as persistent hypercalcemia or arterial calcification, low iPTH levels, or dystrophic bone disease, the use of calcium-based phosphate binders should be restricted (1B).
7. In children with CKD G5D, when the ionized calcium concentration gets < 1.25 mmol/l, individualized adjustments to the calcium-containing dialysate prescription should be considered. Close monitoring of serum calcium levels during treatment is advisable (Not graded).
8. For children with persistent hyperphosphatemia with CKD G5D, implementing an intensified dialysis regimen may be considered to enhance the removal of phosphate from the blood (2D).

### Rationale

Children with CKD require balanced calcium and phosphorus intake to support bone development while preventing ectopic calcification and cardiovascular risks.^43^ Both hypocalcemia and hypercalcemia must be avoided. Serum phosphorus levels should be controlled without dropping below normal ranges to accommodate growing skeletal needs. The 2017 KDIGO guidelines recommend maintaining age-appropriate normal serum calcium and phosphorus levels for pediatric patients with CKD G2 to G5D, though evidence gaps exist for age-specific reference ranges.^7^ Although the Kidney Disease Outcomes Quality Initiative (KDOQI)^11^ guidelines provide age-stratified targets, this protocol primarily adopts KDOQI guidelines recommendations, advocating for age-adjusted normal calcium/phosphorus ranges (Table 3).

**Table 3:** Reference range of serum calcium and phosphorus for children with CKD G2–G5D at different ages

Parameter	0–5 mos	6–12 mos	1–5 yrs	6–12 yrs	13–20 yrs
Total calcium, mg/dl	8.7–11.3	8.7–11.0	9.4–10.8	9.4–10.3	8.8–10.2
Phosphorus, mg/dl	5.2–8.4	5.0–7.8	4.5–6.5	3.6–5.8	2.3–4.5

CKD, chronic kidney disease.

Note: Ca: 1mg/dl = 0.25 mmol/l; P: 1 mg/dl = 0.3229 mmol/l.

Children with CKD G2 to G5D exhibiting low serum calcium should increase intake of high-bioavailability calcium sources such as dairy (e.g., milk, yogurt, and cheese), soy products, and specific vegetables (e.g., Chinese cabbage and broccoli).^44^, ^45^, ^46^, ^47^ Dairy's calcium absorption (∼30%) is higher than that of grains and most vegetables; however, phytate-rich foods (bran grains) would inhibit calcium absorption.^45^^,^^46^ The factors enhancing (e.g., lactose) or impairing (e.g., fiber, phytic/oxalic acids) calcium absorption should also be considered.^45^ When consuming high-calcium dairy, the phosphorus content in the food should be taken into account. Although dietary phosphorus restriction is primary for managing CKD-MBD, overly limiting phosphorus-rich foods (e.g., meat, fish, nuts, and whole grains) may lead to severe malnutrition, because they contain many other important nutrients. And studies show that strict restrictions on dietary phosphorus intake may exacerbate osteomalacia in children with moderate to severe CKD.^47^, ^48^, ^49^ Therefore, serum phosphorus, calcium, and iPTH levels require integrated assessment. Lacking age-specific evidence for calcium or phosphorus intake limits, this guideline adopts KDOQI.^11^ and the Pediatric Renal Nutrition Taskforce^13^ guidelines recommendations with expert consensus: total calcium intake (diet + phosphate binders) should be 100% to 200% of age-adjusted DRI, whereas daily phosphorus intake for children with CKD G3a to G5D should not exceed age-specific DRI, or be <80% DRI if serum phosphorus/iPTH are elevated (Table 4). However, in the future, more high-quality clinical trials are still needed for further confirmation.

**Table 4:** Phosphorus intake in children with CKD G2-G5D at different ages(mg/d)

Serum PTH and phosphorus	0–6 mos	7–12 mos	1–3 yrs	4–8 yrs	9–18 yrs
High PTH and normal phosphorus	≤ 100	≤ 275	≤ 460	≤ 500	≤ 1250
High PTH and high phosphorus	≤ 80	≤ 220	≤ 370	≤ 400	≤ 1000

CKD, chronic kidney disease; PTH, parathyroid hormone.

For pediatric patients with CKD with persistent hypocalcemia unresponsive to dietary control, calcium supplementation should be considered. An Indian trial (*n* = 100 children with nephrotic syndrome) showed combined calcium and vitamin D3 supplementation significantly increased serum calcium (8.5 ± 0.07 vs. 8.7 ± 0.10 mEq/l, *P* = 0.007) and lumbar spine BMD (0.561 ± 0.01 vs. 0.607 ± 0.013 g/cm^2^, *P* < 0.0001) over 1.5 years, with sustained treatment improving BMD Z-scores (*P* = 0.008).^50^ Adult CKD studies showed that calcium supplementation can raise serum calcium levels, lower serum phosphate levels (*P* < 0.001), and increase vertebral BMD (*P* = 0.02–0.05) versus controls^51^^,^^52^ (Supplementary Tables S26–S30). Although KDIGO advises individualized serum calcium management in adults to avoid hypercalcemia, it recommends maintaining age-appropriate serum calcium in pediatric CKD.^7^ Calcium supplementation effectively corrects hypocalcemia, improves bone abnormalities, and mitigates growth impairment in children. However, combining calcium with active vitamin D significantly increases the risk of hypercalcemia, necessitating close monitoring.^53^ Current evidence on pediatric CKD-MBD and calcium remains limited, warranting further research to confirm therapeutic safety and efficacy.

For pediatric patients with CKD with persistent hyperphosphatemia unresponsive to dietary control, phosphate binders are essential. Commonly used phosphate binders in clinical practice include calcium-based phosphate binders and noncalcium-based phosphate binders, which can be further classified into metal-based and nonmetal-based phosphate binders. Calcium-based binders (e.g., calcium carbonate) can effectively lower serum phosphorus (MD = 0.23 mg/dl, 95% confidence interval [CI]: 0.04–0.42 mg/dl) and iPTH (MD = 56 pg/ml, 95% CI: 26–84 pg/ml) but increase hypercalcemia risk (risk ratio: 0.45, 95% CI: 0.35–0.59).^54^ As a typical drug of metal-based binders, lanthanum carbonate can also reduce serum phosphorus (0.55 ± 0.10 vs. 0.18 ± 0.13 mg/dl, *P* = 0.02) with low risk of hypercalcemia.^55^ Iron-based binders can lower serum phosphorus (MD = −2.43 mg/dl, *P* < 0.01) and improve anemia.^56^ Aluminum binders have largely been phased out of long-term clinical use because of skeletal and central nervous system toxicity.

Nonmetal-based phosphate binders are mainly represented by sevelamer. Like calcium-based phosphate binders, sevelamer can effectively lower mean serum phosphorus (0.17 mg/dl, 95% CI: 0.37–0.71 mg/dl), mean calcium-phosphorus product (−1.12 mg/dl, 95% CI: −5.88 to 3.64 mg/dl), and mean serum calcium (−0.40 mg/dl, 95%CI −1.16 to 0.36 mg/dl).^57^ In addition, it is reported that sevelamer can improve the levels of fetuin-A and blood flow–mediated dilation index, reducing the risk of cardiovascular events.^58^ Newer agents (e.g., colestilan, tenapanor, and nicotinamide) are also used to lower serum phosphorus in patients with CKD.^59^, ^60^, ^61^

For growing children with CKD G3a to G5D, calcium-based phosphorus binders are preferred to correct hypocalcemia and support growth (Supplementary Tables S31–S35) If the patient has persistent hypercalcemia, arterial calcification, low iPTH levels, or dynamic bone disease, the use of calcium-based phosphate binders should be restricted.

Besides diet and drugs, dialysis also affects serum calcium and phosphorus levels. Dialysate calcium concentration significantly impacts serum calcium levels and bone density in pediatric patients with CKD G5D. Adult studies indicate low-calcium dialysate (1.25 mmol/l) correlates with lower serum calcium and accelerated bone loss versus higher concentrations (1.50–1.75 mmol/l).^62^^,^^63^ KDOQI guideline^64^ recommended using 1.25 mmol/l dialysate with the use of calcium-based phosphate binders and active vitamin D analogs; however the KDIGO guideline^7^ updated this to 1.25 to 1.50 mmol/l (evidence grade 2C). Children require a positive calcium balance for growth; thus, dialysate calcium must be individualized, considering age, dialysis modality, diet, and medications. For refractory hyperphosphatemia, intensified dialysis regimens—short daily dialysis,^65^^,^^66^ nocturnal intermittent dialysis,^67^^,^^68^ or daily nocturnal dialysis^69^^,^^70^can be used. Studies show that intensified dialysis significantly reduces serum phosphorus (2.14 vs. 1.37 mmol/l, *P* < 0.05), PTH levels, and Ca×P product while increasing Kt/V (1.74 vs. 2.15), allowing reduced or discontinued phosphate binder use.^71^ However, intensified dialysis is associated with higher costs, longer treatment times, challenges in the long-term maintenance of arteriovenous fistulas, and limited accessibility. Current evidence is based on small observational studies, necessitating further validation (Supplementary Tables S36–S39)

### Section 5: Assessment and Management of Parathyroid Function

1. We recommend that the serum 25(OH)D concentration for children with CKD G2 to G5D should be maintained above 30 ng/ml (> 75 nmol/l) (not graded).
2. We recommend supplementing natural vitamin D for children with CKD G2 to G5D who have serum 25(OH)D levels < 30 ng/ml, and selecting the appropriate regimen based on age and severity of deficiency (not graded).
3. We suggest for children with CKD G2-G3, it is recommended to control iPTH levels within the normal range. For children with CKD G4, iPTH levels should be controlled below twice the upper limit of the normal range (ULN) . For children with CKD G5 to G5D, it is recommended to maintain iPTH levels between 2 to 9 times the ULN (not graded).
4. For children with CKD G3-G5D who have severe and progressive secondary hyperparathyroidism (SHPT), we recommend starting initial oral calcitriol therapy to reduce excessively high iPTH levels (1B).
5. When the total dose of weekly oral calcitriol is consistent, oral calcitriol can be administered daily or intermittently, with an initial dose of 10 to 20 ng/kg/d, to control iPTH levels within a reasonable range (1B).
6. For the children with mild SHPT or stable iPTH levels in CKD G3 to G5D, we recommend oral treatment with vitamin D analogues. For patients with CKD, we suggest oral administration of alfacalcidol (1C) and paricalcitol (1B) to control iPTH levels.
7. The combination of cinacalcet, calcitriol, or vitamin D analogues could be considered for children with CKD G4 to G5D who have persistent iPTH levels > 300 pg/ml to effectively control iPTH within the target range (2C).
8. The treatment for children with CKD G4 to G5D and persistently elevated iPTH levels (> 300 pg/ml) could initiate cinacalcet therapy at a low dose (≤ 0.20 mg/kg/d), with subsequent dosage adjustments based on treatment efficacy, up to a maximum daily dose of 60 mg (2C).

### Rationale

Pediatric patients with CKD require serum 25(OH)D > 30 ng/ml (> 75 nmol/l) to mitigate vitamin D deficiency risks and reduce rickets or renal osteodystrophy incidence, according to the European Society for Paediatric Nephrology guideline,^12^ American Society of Endocrinology guideline,^72^ and KDOQI guidelines.^11^ When the serum 25(OH)D level is < 30 ng/ml, it indicates vitamin D insufficiency; when the level is between 5 and 20 ng/ml, it indicates vitamin D deficiency; and when it is < 5ng/ml, it indicates severe vitamin D deficiency. Vitamin D supplementation can help maintain calcium-phosphorus balance, improve bone disease, and delay SHPT without significant hypercalcemia risk.^73^ The benefits of vitamin D supplementation outweigh costs, with high feasibility even in resource-limited settings. This guideline prioritizes vitamin D3 (cholecalciferol) supplementation for its superior efficacy in raising 25(OH)D levels in both healthy and hemodialysis populations.^74^^,^^75^ Supplementation should be individualized by age and deficiency severity (Table 5), followed by regular biochemical monitoring to sustain target levels and minimize complications.

**Table 5:** Supplemental doses of vitamin D for different age groups with vitamin D deficiency in CKD G2-G5D

Age	Loading regimens			Maintenance regimens
	< 12 nmol/l	12–50 nmo/l	50–75 nmol/l	
< 1 yr	600 IU/d	600 IU/d	600 IU/d	400 IU/d
> 1 yr	8000 IU/d	4000 IU/d	2000 IU/d	1000–2000 IU/d based on CKD stage

CKD, chronic kidney disease.

There is no consensus on the recommended range of iPTH levels in children with CKD; however, the existing guidelines agree that the recommended range in children with CKD G2 and G3 is to maintain them within the ULN.^8^^,^^11^ For children with CKD G4, KDOQI guideline^11^ suggests that iPTH can be maintained between 70 and 110 pg/ml whereas the Japanese Society for Dialysis Therapy guideline^9^ recommends controlling it <1.5 ULN. For children with CKD G5/G5D, the European Pediatric Dialysis Working Group guideline^8^ proposes maintaining PTH levels between 2 and 3 ULN, KDOQI guideline^11^ recommends a target range of 200 to 300 pg/ml, KDIGO guideline^7^ advises a target range of 2 to 9 ULN (120–540 pg/ml), and the Japanese Society for Dialysis Therapy guideline^9^ suggests 1.5 to 4.5 × ULN—all primarily based on expert consensus with limited evidence. This guideline synthesizes recommendations as follows: maintain iPTH within normal range for CKD G2 and G3, < 2 ULN for CKD G4, and between 2 and 9 ULN for CKD G5/G5D, for balancing biological function, reducing bone disease incidence, mortality risk, and quality of life.

Elevated iPTH is common in children with CKD, primarily associated with factors such as reduced calcitriol, hypocalcemia, and hyperphosphatemia.^76^ Active vitamin D and its analogs can effectively lower iPTH and improve bone metabolism. A multicenter RCT study showed that 52% of children in the calcitriol group achieved a ≥30% reduction in PTH (vs. 19% in the placebo group).^77^ An RCT study confirmed no significant difference in efficacy between daily and intermittent oral calcitriol^78^^,^^79^ (Supplementary Tables S40–S44) Among vitamin D analogs, Finnish and Japanese studies both reported that the use of alfacalcidol can help significantly reduce serum iPTH levels in children with CKD with concurrent SHPT.^80^^,^^81^ A systematic review demonstrated that the use of paricalcitol significantly reduced iPTH (odds ratio = 0.12; 95% CI: 0.05–0.29; *P* < 0.001) with minimal impact on serum calcium and phosphorus.^82^ (Supplementary Tables S45–S49) Guidelines recommend using active vitamin D or analogs for children with CKD stages G3a to G5 and SHPT. The initial dose of oral calcitriol is 10 to 20 ng/kg/d (oral/i.v. infusion equally effective, intermittent dosing suitable for patients with poor compliance). The recommended dose for alfacalcidol in clinical practice is 0.5 μg/d for children. And the recommended initial dosage of paricalcitol in children with CKD is 0.04 μg/kg/d (oral administration is more feasible). Treatment requires an individualized approach, with close monitoring of serum calcium, phosphorus, and iPTH levels; and dosage adjustments should be based on SHPT severity and the magnitude of iPTH reduction.

For pediatric patients with CKD with SHPT, persistently elevated iPTH levels require effective intervention. Cinacalcet, a common calcimimetic, has demonstrated efficacy in multiple studies. A phase 3 trial in Europe and the USA showed that 54.5% of dialysis children in the cinacalcet group achieved ≥ 30% iPTH reduction after 14 months of treatment (vs. 19% in controls).^83^ A cohort study in Saudi Arabia reported that iPTH levels decreased by > 60% from baseline in all children treated with cinacalcet (60%–97%).^84^ A US case series study showed a significant reduction in mean iPTH levels after cinacalcet treatment (929 pg/ml [interquartile range: 572–1056 pg/ml] vs. 385 pg/ml [interquartile range:140–710 pg/ml], *P* < 0.01)^85^ (Supplementary Tables S50–S54) This agent significantly controls iPTH with good tolerability, but hypocalcemia risk warrants vigilance. Although etelcalcetide showed superior efficacy to cinacalcet in adult studies (odds ratio = 2.78; 95% CI: 1.19–6.67),^86^ pediatric data are limited to a phase 1 trial (transient iPTH decrease after single dose), providing scarce evidence for pediatric use.^87^ Guidelines recommend combining cinacalcet (starting dose ≤ 0.20 mg/kg/d, maximum < 180 mg/d) with vitamin D analogs for children aged ≥ 3 years with CKD stages G4 to G5D and persistently elevated iPTH > 300 pg/ml. Treatment requires strict monitoring of serum calcium (weekly during the first week and titration phase, monthly during maintenance), with dose reduction or discontinuation if hypocalcemic symptoms or excessively low iPTH occur. Current evidence for calcimimetics in children still lacks large-sample, high-quality RCTs.

For pediatric patients with CKD-MBD with medication-refractory SHPT, parathyroidectomy is an effective option. Both a Spanish study^88^ (2018, *n* = 13) and an Argentinian study^89^ (2017, *n* = 15) showed a significant postoperative decrease in PTH (*P* < 0.05) and improvement in bone disease symptoms, though complications such as hungry bone syndrome or hypoparathyroidism may occur.^88^, ^89^, ^90^ However, delicate intraoperative manipulation and timely management through close postoperative observation can prevent complications such as bone starvation syndrome, laryngeal reentrant nerve injury, and wound infection. More research is needed to clarify the surgical indications and optimal surgical methods for children.

While the preceding 5 sections detail management strategies from various dimensions, in Figure 2, we integrate these recommendations into a clear, actionable clinical decision pathway. Designed to assist clinicians in rapidly formulating treatment plans, this flowchart outlines the entire process from screening and diagnostic evaluation to stratified treatment and follow-up monitoring, visually demonstrating the logical application and sequence of the recommendations in daily practice.

**Figure 2:**
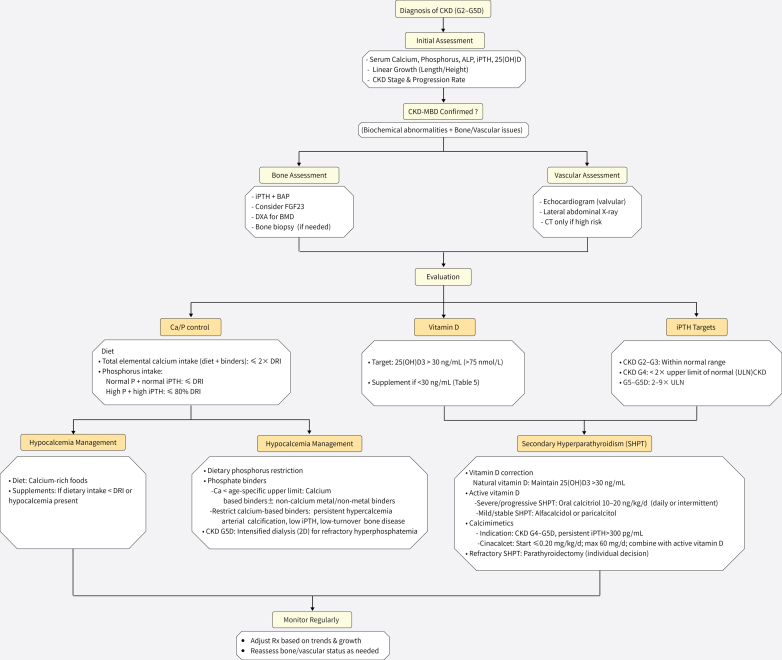
Comprehensive Algorithm for the Diagnosis and Management of CKD-MBD. 25(OH)D_3_, 25-hydroxyvitamin D_3_; ALP, alkaline phosphatase; BAP, bone ALP; BMD, bone mineral density; Ca, calcium; CKD, chronic kidney disease; CT, computed tomography; DRI, Dietary Reference Intakes; DXA, dual-energy x-ray absorptiometry; iPTH, intact parathyroid hormone; MBD, mineral and bone disorder; P, phosphorus; SHPT, secondary hyperparathyroidism.

### Conclusion

This guideline is the first evidence-based consensus for the management of pediatric CKD-MBD in China. Developed following the Grading of Recommendations Assessment, Development, and Evaluation methodology, it systematically reviewed the domestic and international literature to provide actionable recommendations tailored to the national health care landscape. Clinically, this guideline establishes a framework to standardize the screening, diagnosis, and treatment of CKD-MBD in children; however, there are some limitations in this guideline, including a paucity of high-quality pediatric evidence for some clinical questions, limited drug availability for children, and the restricted feasibility of certain recommended tests in primary care settings. Future efforts will focus on facilitating multicenter studies to generate high-level pediatric evidence, thereby enhancing the availability of child-appropriate treatments.

### Research Prospect

Based on the current evidence, the Guideline Development Working Group suggests the following directions for further research in the diagnosis and management of children with CKD-MBD: (ⅰ) to study the accuracy of the combined diagnosis of iPTH and BAP in the diagnosis of bone turnover in children with CKD-MBD, (ⅱ) to continue to study the sensitivity and specificity of echocardiography and lateral abdominal X-ray in the diagnosis of vascular calcification in children with CKD-MBD, (ⅲ) to continue research to explore the therapeutic effect of intensive dialysis in children with CKD-MBD complicated by hyperphosphatemia, (ⅳ) to further analyze the application effect of sevelamer in children with CKD-MBD and compare it with calcium-phosphorus binders, (ⅴ) to further study the target range of iPTH in children with CKD G4 and G5, and (ⅵ) to explore the surgical indications for thyroidectomy in children and the best surgical method for different patients.

This guideline will promote the progress of diagnosis and treatment of children with CKD-MBD and help improve the level of children's health. Aiming to promote the progress of diagnosis and treatment of children with CKD-MBD and help improve the level of children's health, we will conduct more high-level research with colleagues at home and abroad, gradually explore and verify, to build and improve the standard diagnosis and treatment system of children with CKD-MBD, and better contribute to the development of children's health.

## Appendix

### List of the Life Participation Workshop Investigators

Ai-Hua Zhang, Qui Li, Mo Wang, Hong Xu, Hui Wang, Chun-lin Gao, Xiao-rong Liu, Yu-bin Wu, Shu-Zhen Sun, Yuhong Tao, Xiao-shan Shao, Fang Wang, Li-Jun Zhao, Xiao-yun Jiang, Ying-jie Li, Li Yu, Zheng-kun Xia, Xi-qiang Dang, Jian-hua Mao, Jian-hua Zhou, Qian Shen, Wen-yan Huang, Xiao-wen Wang, Ying Shen, Song-ming Huang, Hui-mei Huang, and Dong-feng Zhang.

## Disclosure

All the authors declared no competing interests.
